# Stillbirth, newborn and infant mortality: trends and inequalities in four population-based birth cohorts in Pelotas, Brazil, 1982–2015

**DOI:** 10.1093/ije/dyy129

**Published:** 2019-03-18

**Authors:** Ana M B Menezes, Fernando C Barros, Bernardo L Horta, Alicia Matijasevich, Andréa Dâmaso Bertoldi, Paula D Oliveira, Cesar G Victora, Pelotas Cohorts Study Group, Aluisio J D Barros, Diego G Bassani, Fernando C Wehrmeister, Helen Gonçalves, Iná S Santos, Joseph Murray, Luciana Tovo-Rodrigues, Maria Cecilia F Assunção, Mariangela F Silveira, Marlos Rodrigues Domingues, Pedro R C Hallal

**Affiliations:** 1 Federal University of Pelotas, Brazil; 2 University of Toronto, Canada; 1 Post-Graduate Program in Epidemiology, Federal University of Pelotas, Pelotas, Brazil; 2 Post-Graduate Program in Health and Behavior, Catholic University of Pelotas, Pelotas, Brazil; 3 Department of Preventive Medicine, Faculty of Medicine FMUSP, University of São Paulo, São Paulo, Brazil

**Keywords:** stillbirth, infant mortality, cohort studies, socio-economic factors, infant newborn

## Abstract

**Background:**

Infant-mortality rates have been declining in many low- and middle-income countries, including Brazil. Information on causes of death and on socio-economic inequalities is scarce.

**Methods:**

Four birth cohorts were carried out in the city of Pelotas in 1982, 1993, 2004 and 2015, each including all hospital births in the calendar year. Surveillance in hospitals and vital registries, accompanied by interviews with doctors and families, detected fetal and infant deaths and ascertained their causes. Late-fetal (stillbirth)-, neonatal- and post-neonatal-death rates were calculated.

**Results:**

All-cause and cause-specific death rates were reduced. During the study period, stillbirths fell by 47.8% (from 16.1 to 8.4 per 1000), neonatal mortality by 57.0% (from 20.1 to 8.7) and infant mortality by 62.0% (from 36.4 to 13.8). Perinatal causes were the leading causes of death in the four cohorts; deaths due to infectious diseases showed the largest reductions, with diarrhoea causing 25 deaths in 1982 and none in 2015. Late-fetal-, neonatal- and infant-mortality rates were higher for children born to Brown or Black women and to low-income women. Absolute socio-economic inequalities based on income—expressed in deaths per 1000 births—were reduced over time but relative inequalities—expressed as ratios of mortality rates—tended to remain stable.

**Conclusion:**

The observed improvements are likely due to progress in social determinants of health and expansion of health care. In spite of progress, current levels remain substantially greater than those observed in high-income countries, and social and ethnic inequalities persist.

## Introduction

**Key Messages**

- There were important reductions in fetal and child death rates during 1982–2015: stillbirths fell by 47.8%, neonatal mortality by 57.0% and infant mortality by 62.0%.
- The number of infant deaths in the city fell from 215 in 1982 to 59 in 2015, and the infant-mortality rate fell from 36.4 to 13.8/1000 live births, respectively.
- Deaths due to infectious diseases showed the largest reductions and, in 2015, there were no deaths due to diarrhoea compared with 25 in 1982.
- Absolute socio-economic inequalities in mortality were reduced over time, but relative inequalities tended to remain stable.

Infant-mortality rates for Brazil have declined by 80.4% in the last three decades, from 71.3 per 1000 live births in 1982 to 14.0 in 2015.^1^ As a consequence, Brazil reached the fourth Millennium Development Goal, which required a two-thirds reduction between 1990 and 2015 of the mortality of children aged under 5 years.^2–4^

Neonatal mortality also fell by 63.4%, from 33.4 to 8.2 deaths per 1000 between 1982 and 2015, therefore showing a slower rate of reduction than infant or under-5 mortality. Among neonatal deaths, the reduction was faster for late-neonatal (7–28 days) than for early-neonatal (0–6 days) mortality.^5^ These findings are consistent with information on time trends in causes of death, which show a marked decline in infectious diseases, coupled with a relative increase in the proportion of deaths due to complications of prematurity.^6^^,^^7^

Unlike data on neonatal, infant and under-5 mortality, which are widely available from vital registration or demographic surveys, few low- and middle-income countries (LMICs) are able to measure fetal mortality, and thus existing estimates are largely based on modelling. Blencowe *et al.*^8^ estimated that the late-fetal-mortality rate (28 weeks’ gestation or more) for the whole of Latin America fell from 11.3 per 1000 total births in 1982 to 8.2 in 2015—a reduction of 27.4%. A systematic review of fetal mortality in Brazil showed poor quality in the routine information system, with low coverage of the information system and—even when records were present—a high frequency of missing information on causes of death and maternal characteristics. Most deaths that were reported fell into the antepartum category.^9^

The important decline in child mortality in Brazil was accompanied by reductions in geographic and socio-economic inequalities,^7^ but these are still far from being eliminated. In particular, mortality rates for children living in urban slums or for indigenous children still remain well above those among the rest of the population.^10^

In order to test the hypothesis that mortality rates are falling and that inequalities are being reduced, we report on the levels and causes of late-fetal, newborn and infant mortality in the city of Pelotas in Southern Brazil, where four population-based birth cohort studies were carried out in the years of 1982, 1993, 2004 and 2015. The existence of four comparable studies over more than three decades is unique and provides the opportunity of studying not only overall trends, but also trends in socio-economic inequalities, which is not possible to do using other data sources.

Earlier publication compared trends up to the 2004 cohort.^11^ We focus on how mortality levels and the degree of socio-economic inequalities have evolved over more than three decades.

## Methods

Each cohort recruited all hospital births that occurred in the calendar years of 1982, 1993, 2004 and 2015. A surveillance system was set up to detect all infant deaths. In the first two cohorts, there was evidence that the coverage of death registration in the city was incomplete. Therefore, during 1982–83 and 1993–94, our research team made fortnightly visits to all emergency departments, paediatric wards and intensive-care units, cemeteries and the city’s vital registration office. We found that 24% of the deaths to children born in 1982 and 5.4% of those born in 1993 had not been registered.^12^ In 2004 and 2015, the vital registration system had full coverage and a municipal audit committee for infant deaths was in place, so that it was not necessary to set up our own surveillance system. Once deaths were identified, we obtained copies of the death certificates and reviewed hospital case notes. If necessary, we interviewed the paediatricians and obstetricians (for late-fetal deaths), as well as the child’s parents.

Late-fetal deaths were classified as antepartum or intrapartum; there was no attempt to identify the causes of death. In 1982 and 1993, the analyses of fetal deaths were restricted to fetuses with gestational age of 28 or more weeks and/or a weight of 1000 g or more. In 2004 and 2015, information was also collected on fetal deaths occurring between 20 and 27 weeks’ gestation and/or a weight of 500 g or more, but the analyses presented here is based on ≥28 weeks’ gestation. In 1982 and 1993, gestational age was based on the date of the last menstrual period provided by the mother whereas, in 2004 and 2015, we adopted the best obstetric estimate, based primarily on ultrasound and secondarily on the last menstrual period, when ultrasound information was not available. Birthweights were measured using paediatric scales with a precision of 100 g that were regularly calibrated by the research team. Details on the assessment of gestational age and birthweight in the four cohorts are available elsewhere.^13^^,^^14^

Causes of infant deaths were classified independently by two physicians based on the death certificates, hospital case notes and, when applicable, the notes from interviews with doctors and families. When there was disagreement between the two reviewers, a senior paediatrician (F.B.) acted as an arbiter to establish the cause. In 1982, because information on causes of death was often poor, deaths were grouped according to the Wigglesworth classification, with slight modifications.^15^ The following groups were used: perinatal causes, malformations, diarrhoea, respiratory-tract infections, other infections and other causes; the latter also included ill-defined causes. In 1993, ICD9 was used to classify causes of death and, in 2004 and 2015, the ICD10 classification was used.^15–17^ For consistency with the 1982 cohort results, the same groups of causes were used for the later cohorts.

The following mortality rates were calculated, with their respective 95% confidence intervals (CIs), using standard international definitions.^18^^,^^19^ Late-fetal- and perinatal-mortality rates were expressed for 1000 total births; all other rates had 1000 live births as the denominator. Analyses of mortality rates were stratified by family-income terciles, maternal skin colour (White, Brown or Black) and sex of the child. Analyses according to family-income quintiles were carried out initially but, due to the small number of deaths in some categories, this variable was re-coded into terciles. Further information on the stratification variables is available elsewhere.^13^

Data analyses included chi-squared tests for heterogeneity and linear trends. Poisson regression with robust variance was used to analyse ratios of mortality rates according to the categories of explanatory variables across the four cohorts, when there was no statistical evidence of an interaction with the cohort year.^20^ If there was an interaction, we present the rate ratios separately for each cohort. Prevalence ratios for neonatal and infant mortality in the 1993, 2004 by and 2015 cohorts, using the 1982 cohort as reference, were evaluated through Poisson regression adjusted for gestational age.

The slope index of inequality and concentration index were used to assess income-related disparities.^21^ The slope index (SII) represents the absolute difference in the fitted value of the health indicator between the highest (score of 1) and the lowest (score of 0) values of the socio-economic indicator rank; it is expressed in percentage points. The concentration index (CIX) is expressed on a scale from –100 to +100, with zero representing equal distribution of the attribute across the wealth scale. Positive values indicate that the outcome is more common among the rich, whereas negative values indicate higher levels among the poor.^21^ Stata software 13 was used for the analyses.^22^

Ethical approval for studies was not required in Brazil until 1996. The 2004 study was approved by the Ethics Committee of the School of Medicine and the 2015 by the School of Physical Education, Federal University of Pelotas, and written consent was obtained from the mothers. All datasets were anonymized for the present analyses.

## Results

The number of live births fell by nearly one-third from 1982 to 2015 (Table 1). All death rates were reduced, except for late-neonatal deaths, for which there was no statistical evidence of a decline. The late-fetal-death rate (≥28 weeks’ gestation) fell by 47.8% from 16.1 to 8.4 per 1000 total births. In 2004 and 2015, it was also possible to calculate the fetal-death rates starting at a gestational age of 20 weeks, which were equal to 13.1 (95% CI 9.7–16.40) in 2004 and 12.5 (95% CI 9.2–15.8) in 2015. The antepartum late-fetal-death rate fell from 13.1 (10.3–16.0) to 8.1 (5.4–10.8) from 1982 to 2015—a reduction of 38.2%. In contrast, intrapartum deaths fell by 90% from 2.5 to 0.2 per 1000 from 1982 to 2004, when only one such death took place. Neonatal mortality fell from 20.1 to 8.7—a reduction of 57.0%. Infant mortality fell by 62.0%, from 36.4 to 13.8 (Table 1), whereas the absolute number of infant deaths dropped from 215 to 59 in the city.

**Table 1.** Late-fetal-, perinatal-, neonatal- and infant-mortality rates in four birth cohorts, Pelotas, Brazil

	Birth cohorts (years)				
	1982	1993	2004	2015	*p*
Number of births*	6011	5304	4272	4311	–
Number of live births	5914	5249	4231	4275	–
Late-fetal deaths*					
Number	97	55	41	36	<0.001
Fetal-mortality rate^a^ (1000 total births)	16.1	10.4	9.6	8.4	
95% CI	13.0–19.3	7.6–13.1	6.7–12.5	5.6–11.0	
Early-neonatal deaths					<0.001
Number	97	62	38	27	
Early-neonatal-mortality rate^b^ (1000 live births)	16.4	11.8	9.0	6.3	
95% CI	13.0–19.5	8.9–14.7	6.2–11.8	3.9–8.7	
Perinatal deaths (fetal* + early-neonatal)					–
Number	194	117	79	63	<0.001
Perinatal-mortality rate (1000 total births)	32.3	22.1	18.5	14.6	
95% CI	27.7–36.5	18.1–26.0	14.5–22.5	11.0–18.2	
Neonatal deaths					<0.001
Number	119	75	52	37	
Neonatal-mortality rate (1000 live births)^c^	20.1	14.3	12.3	8.7	
95% CI	16.5–23.7	11.1–17.5	9.0–15.6	5.8–11.4	
Late-neonatal deaths					0.317
Number	22	13	14	10	
Late-neonatal-mortality rate (1000 live births)^d^	3.7	2.5	3.3	2.3	
95% CI	2.2–5.3	1.1–3.8	1.6–5.0	0.9–3.8	
Infant deaths					<0.001
Number	215	111	82	59	
Infant-mortality rate (1000 live births)^e^	36.4	21.1	19.4	13.8	
95% CI	31.6–41.1	17.3–25.0	15.2–23.5	10.3–17.3	

*Gestational age ≥28 weeks; CI, confidence interval; total number of births considering gestational age ≥20 weeks is 4287 (2004) and 4329 (2015).

^a^Total stillbirths/total births.

^b^Total deaths before 7 full days of life/total births.

^c^Deaths occurred from the first day to 28 incomplete days after birth/total live births.

^d^Deaths occurred after 7 full days and before 28 full days of life/total live births.

^e^Deaths during the first year of life/total live births.

*p*-value: χ^2^ for trend.

Tables 2–4 show late-fetal-, neonatal- and infant-mortality rates according to sex, maternal skin colour and family income in each cohort. The supplementary materials (Supplementary Tables 1–3, available as Supplementary data at *IJE* online) include the absolute numbers of deaths for all analyses reported in this paper, whereas results for perinatal mortality are shown in Supplementary Table 4 and Supplementary Table 4a, available as Supplementary data at *IJE* online, and those for post-neonatal mortality in Supplementary Table 5 and Supplementary Table 5a, available as Supplementary data at *IJE*online. Trends in absolute inequalities are described at the end of this section.

Table 2 shows late-fetal-mortality rates in each cohort according to the explanatory variables. The pooled mortality sex ratio was equal to 1.07 (95% CI 0.82–1.39) for boys relative to girls, and there was no evidence that the ratio changed over time (*P* = 0.48 for the interaction with cohort year). In contrast, there was statistical evidence (*P* = 0.013) that late-fetal mortality according to maternal skin colour changed over time: the ratios for Black or Brown skin colour, relative to White, were 1.26 (95% CI 0.78–2.03), 1.93 (1.12–3.34), 2.31 (1.26–4.26) and 3.16 (1.64–6.08) in 1982, 1993, 2004 and 2015, respectively. Late-fetal-mortality rates remained stable at around 20 per 1000 for Black women, but fell by two-thirds for White mothers.

**Table 2.** Late-fetal-mortality rate (gestational age ≥28 weeks) according to sex, maternal skin colour and socio-economic status in four birth cohorts, Pelotas, Brazil

	Late-fetal-mortality rate per 1000 total births (95% CI)				
	1982	1993	2004	2015	p*
Sex	*p* = 0.950	*p* = 0.612	*p* = 0.578	*p* = 0.210	
Males	15.9 (11.5–20.3)	10.3 (6.4–14.1)	8.1 (4.4–11.9)	10.0 (5.9–14.2)	0.024
Females	16.1 (11.5–20.6)	9.0 (5.4–12.6)	9.7 (5.5–14.0)	6.6 (3.1–10.0)	0.002
Maternal skin colour	*p* = 0.345	*p* = 0.044	*p* = 0.018	*p* < 0.001	
White	15.4 (12.0–18.9)	8.6 (5.7–11.4)	7.1 (4.1–10.0)	5.2 (2.6–7.7)	<0.001
Brown	19.4 (11.2–27.7)^a^	12.7 (0.0–26.9)	13.4 (0.3–26.4)	10.6 (2.1–18.9)	0.756
Black		17.5 (9.2–25.7)	17.4 (8.7–26.2)	21.4 (10.3–32.6)	0.856
Family income (tertiles)	*p* < 0.001*	*p* = 0.653*	*p* = 0.135*	*p* = 0.008*	
T1 (poorest)	34.4 (26.5–42.3)	10.9 (5.7–16.1)	9.7 (4.6–14.7)	12.4 (6.8–17.9)	<0.001
T2	9.0 (4.9–13.1)	11.0 (6.2–15.8)	15.0 (8.6–21.3)	8.9 (3.9–13.9)	0.651
T3 (richest)	4.5 (1.6–7.5)	9.4 (4.9–13.8)	4.2 (0.8–7.6)	3.5 (0.4–6.6)	0.398

*p*-value: χ^2^ test for heterogeneity.

*p-value: χ^2^ for trend.

^a^Black and Brown were combined.

Regarding family income (Table 2), the lowest mortality rates in all cohorts were observed in the richest tercile, for which the rate was already low (4.5 per 1000) in 1982. Otherwise, patterns were not very clear, with the ratios between the poorest and richest terciles being greater than three times in the first and last cohorts, and smaller in 1993 and 2004. The only group with a substantial decline in mortality was the poorest tercile.

Neonatal-mortality rates are shown in Table 3. The associations between mortality and sex, skin colour and income did not change over time; interaction terms with cohort year had *P*-levels of 0.79, 0.56 and 0.46, respectively. Thus, results for the four cohorts were pooled. Newborn deaths were 1.44 (95% CI 1.13–1.82) times more common among boys than girls and 1.49 (1.16–1.91) times higher for children born to Black or Brown mothers than for those born to White women. Regarding family income, pooled rates in the poorest tercile were 1.96 (1.44–2.67) times higher than in the richest tercile; the rate ratio between the middle and richest terciles was 1.69 (1.23–2.32).

**Table 3.** Neonatal-mortality rate according sex, maternal skin colour and socio-economic status in four birth cohorts, Pelotas, Brazil

	Neonatal-mortality rate per 1000 live births				
	1982	1993	2004	2015	p*
Sex	*p* = 0.169	*p* = 0.029	*p* = 0.093	*p* = 0.453	
Males	22.4 (17.1–27.7)	17.7 (12.6–22.7)	15.0 (9.9–20.1)	9.7 (5.6–13.8)	<0.001
Females	17.4 (12.6–22.2)	10.6 (6.7–14.5)	9.3 (5.2–13.5)	7.6 (3.9–11.3)	0.001
Maternal skin colour	*p* = 0.521	*p* = 0.068	*p* = 0.061	*p* = 0.261	
White	19.6 (15.7–23.5)	12.3 (8.9–15.7)	10.0 (6.5–13.5)	7.5 (4.4–10.5)	<0.001
Brown	22.6 (13.7–31.6)^a^	25.6 (5.3–45.9)	13.6 (0.3–26.8)	8.9 (1.1–16.7)	0.265
Black		19.9 (11.0–28.8)	20.1 (10.6–29.6)	14.1 (4.9–23.2)	0.074
Family income (tertiles)	*p* = 0.008*	*p* = 0.014*	*p* = 0.094*	*p* = 0.1061*	
T1 (poorest)	23.9 (17.2–30.7)	18.8 (12.0–25.5)	16.8 (10.1–23.4)	10.5 (5.4–15.7)	0.003
T2	24.3 (17.5–31.0)	14.5 (8.9–19.9)	10.1 (4.9–15.4)	10.5 (5.0–15.9)	0.001
T3 (richest)	12.2 (7.3–17.0)	8.9 (4.6–13.2)	9.9 (4.7–15.0)	4.9 (1.3–8.6)	0.050

*p*-value: χ^2^ test for heterogeneity.

*p-value: χ^2^ for trend.

^a^Black and Brown were combined.

Regarding infant mortality (Table 4), rates were 1.21 (1.10–1.45) higher for boys than for girls, with no evidence of change over time (*P* = 0.58 for interaction with cohort year). There was also no evidence of a change in the ethnic gradient over time (*P* = 0.97), with children born to Black or Brown mothers showing 1.76 (1.45–2.13) times higher risk than those born to White mothers. For income, the pooled ratio of mortality in the poorest relative to the richest tercile was 3.11 (2.42–4.01) and that between the middle and richest tercile 1.85 (1.41–2.43). There was no interaction between income and cohort year (*P* = 0.25).

**Table 4.** Infant-mortality rate according sex, maternal skin colour and socio-economic status in four birth cohorts, Pelotas, Brazil

	Infant-mortality rate per 1000 live births (95% CI)				
	1982	1993	2004	2015	p*
Sex	*p* = 0.260	*p* = 0.044	*p* = 0.322	*p* = 0.820	
Males	38.9 (32.0–45.7)	25.0 (19.0–31.0)	21.4 (15.3–27.5)	13.3 (8.6–18.2)	<0.001
Females	33.4 (26.8–39.9)	17.0 (12.1–21.9)	17.2 (11.5–22.9)	14.2 (9.2–19.3)	<0.001
Maternal skin colour	*p* < 0.001	*p* = 0.013	*p* = 0.008	*p* = 0.068	
White	32.2 (27.2–37.1)	18.0 (13.9–22.1)	15.5 (11.2–19.9)	11.7 (7.9–15.5)	<0.001
Brown	55.7 (41.8–69.5)^a^	34.2 (10.9–57.5)	23.7 (6.3–41.1)	14.3 (4.4–24.1)	0.069
Black		31.4 (20.3–42.4)	31.9 (20.1–43.8)	23.5 (11.7–35.2)	0.001
Family income (tertiles)	*p* < 0.001*	*p* < 0.001*	*p* = 0.002*	*p* = 0.009*	
T1 (poorest)	61.6 (51.0–72.3)	30.5 (21.9–39.0)	28.7 (20.0–37.3)	19.1 (12.2–26.0)	<0.001
T2	30.8 (23.2–38.4)	23.4 (16.4–30.3)	16.7 (9.9–23.4)	14.2 (7.9–20.5)	<0.001
T3 (richest)	16.7 (11.1–22.4)	9.5 (5.0–13.9)	12.7 (6.9–18.5)	7.8 (3.2–12.3)	0.040

*p*-value: χ^2^ test for heterogeneity.

*p-value: χ^2^ for trend.

^a^Black and Brown were combined.

Because of the important increase in preterm deliveries during the study period, we assessed the estimated change in neonatal and infant mortality, adjusting for the observed trends in gestational age distribution through Poisson regression (Supplementary Table 6, available as Supplementary data at *IJE* online). In the unadjusted analyses, the neonatal mortality in 2015 was equal to 43% (prevalence ratio of 0.43) of the level observed in 1982. After adjustment for the gestational age distribution, the reduction was even sharper: mortality in 2015 was equal to 33% of the 1982 rate. Similar results were obtained for infant mortality, with prevalence ratios of 0.38 in the unadjusted analyses and 0.30 after adjustment.

Income-related inequalities in mortality were assessed through the slope and concentration indices. For late-fetal mortality, the slope index declined from –47.3 (95% CI −62.0 to –32.5) deaths per 1000 total births in 1982 to –13.3 (–23.2 to –3.4) in 2015, showing a marked reduction in absolute inequalities. There was not much evidence of a change in relative inequalities, as the concentration index was equal to –42.9 (–53.3 to –32.5) in 1982 and –18.5 (–35.9 to –10.9) in 2015, but the CIs overlapped. None of the two indices presented important changes for neonatal mortality; the slope index was equal to –17.4 (–29.9 to –5.0) in 1982 and –8.3 (−17.9 to 13.2) in 2015, and the concentration index –11.0 (–20.0 to –2.0) and –19.0 (–36.1 to –1.8), respectively. Lastly, absolute inequalities in infant mortality were markedly reduced with slope indices of –68.7 (–88.4 to –49.0) in 1982 and –17.1 (–30.0 to –4.3) in 2015, but relative inequalities remained almost unchanged with concentration indices of –25.0 (–31.8 to –18.2) and –24.3 (–38.0 to –10.7), respectively. These results for relative inequalities, based on the concentration index, are consistent with the absence of an interaction between income and cohort year in the multiplicative Poisson models.

Table 5 shows important reductions in the absolute number of causes of death, as well as mortality rates, for all causes. From 1982 to 2015, mortality rates fell by 47% for perinatal causes and 64% for malformations. Diarrhoea deaths were eliminated, and there were reductions of 79% for respiratory infections and 87% for other infections. Other or ill-defined causes, including sudden infant deaths, fell by 51%.

**Table 5.** Number of deaths and cause-specific infant-mortality rates 1982–2015

	1982	1993	2004	2015	
	*N* rate per 1000 live births (95% CI)	*N* rate per 1000 live births (95% CI)	*N* rate per 1000 live births (95% CI)	*N* rate per 1000 live births (95% CI)	*p*
Perinatal	92	57	41	36	0.001
	15.6 (12.4–18.7)	10.9 (8.1–13.7)	9.7 (6.7–12.6)	8.4 (5.7–11.2)	
Congenital malformations	27	25	8	7	0.002
	4.6 (2.8–6.3)	4.8 (2.9–6.6)	1.9 (0.6–3.2)	1.6 (0.4–2.8)	
Diarrhoea	25	9	1	0	<0.001
	4.2 (2.6–5.9)	1.7 (0.6–2.8)	0.2 (0.0–0.7)	0.00	
Respiratory infections	25	7	13	4	0.007
	4.2 (2.6–5.9)	1.3 (0.3–2.3)	3.1 (1.4–4.7)	0.9 (0.0–1.9)	
Other infections	18	1	5	2	0.002
	3.0 (1.6–4.4)	0.2 (0.0–0.6)	1.2 (0.1–2.2)	0.5 (0.0–1.1)	
Other causes or ill-defined	28	12	14	10	0.069
	4.7 (3.0–6.4)	2.3 (1.0–3.6)	3.3 (1.6–5.0)	2.3 (0.9–3.8)	
Total	215	111	82	59	

*p*-value: χ^2^ for trend.

## Discussion

During the study period, all-cause and cause-specific mortality rates were reduced; stillbirths fell by 47.8%, neonatal mortality by 57% and infant mortality by 62%. The leading causes of death were perinatal conditions and the largest reductions were observed for infectious diseases. Children born to Brown or Black women and to low-income women showed the highest late-fetal-, neonatal- and infant-mortality rates; although absolute socio-economic inequalities were reduced over time, relative inequalities remained stable.

Few studies on time trends in stillbirths are available from LMICs. Our results suggest that the decline in late-fetal deaths was slightly slower than for neonatal deaths, and markedly slower than for infant deaths as a whole. Much of the decline was due to the virtual elimination of intrapartum deaths—possibly one of the few advantages of having a caesarean-section rate above 60% in 2015.^23^^,^^24^ The 2015 rate of 8.4 stillbirths per 1000 is very similar to that of 8.2 estimated for Latin America as a whole,^8^ but remains over twice as high as the rate of 3.5 per 1000 estimated for high-income countries.^25^ It is striking that, whereas late-fetal mortality fell by more than 60% for gestations of White mothers, it remained stable over time at around 20 per 1000 for Black women. Unfortunately, we have no information on causes of stillbirths. It is well known that congenital syphilis remains a problem in Brazil,^26^^,^^27^ but the Zika virus epidemic never reached Pelotas. In 2015, stillbirths accounted for 38% (36 over 36 plus 59) deaths occurring from 28 weeks’ gestation to the first birthday in Pelotas. Increasing the visibility of stillbirths is essential for raising political awareness of the need to prevent them.^28^

The neonatal-mortality rate in 2015 of 8.7 per 1000 live births was close to the rate of 8.2 estimated for Brazil.^1^ In contrast, the decline observed in Pelotas since 1982 (from 20.1 to 8.7) was much slower than that recorded for the country as a whole (from 33.4 to 8.2).^1^ This is likely related to lower levels in Pelotas in 1982, as well as to the fact that socio-economic standards and availability of health care in Pelotas were substantially ahead of other regions of the country in the 1980s. Over time, previously less developed states caught up in terms of economic growth and of universal access to health care, particularly from 1989 when the Unified Health System was created.^29^ There is evidence of an important increase in preterm deliveries, which has been attributed—at least in part—to the extremely high caesarean-section rates.^7^^,^^30–32^ The preterm epidemic may have contributed to the relatively low rate of decline in neonatal deaths in Pelotas. Our 2015 neonatal-mortality rate is similar to that observed in Latin America as a whole, but still four times larger than that recorded in high-income countries in Western Europe.^1^

Infant mortality in Pelotas fell by 62.0% over the study period, from 36.4 to 13.8 per 1000 live births. The size of this reduction becomes more palpable in absolute numbers: 215 annual deaths in 1982 in a city of 230 000 inhabitants, compared with 59 deaths in 2015 for 340 000 inhabitants; the decline in the number of births, from about 6000 to just over 4000, has also played a role, but the size of the reduction remains impressive. As for neonatal deaths, the 62.0% reduction in infant mortality was slower than that of 80.5% observed for Brazil as a whole. Infant mortality in Pelotas remains about five times higher than in Western Europe, and at similar rates to Latin America and the Caribbean,^1^ though higher than the rates observed in our South American neighbours Uruguay and Argentina—9/1000 and 11/1000, respectively.^1^

Adjustment for the gestational age distribution in each cohort suggested that the declines in neonatal and mortality would be even more marked in the absence of the rising trend in preterm births in the city.^14^

Our data on cause-specific mortality since the 1980s are unique. The data reveal the huge reduction in infectious diseases, in particular the eradication of diarrhoea deaths. This decline is similar to what has been observed over a shorter time period in Brazil.^7^ Our analyses of hospitalizations in the four cohorts^33^ show similar declining trends for infectious diseases.

Our analyses of inequalities confirmed the higher risk of mortality associated with children born to women of low family income and for afro-descendants for late-fetal, neonatal and infant mortality. Boys were at higher risk of neonatal and infant mortality, but not of late-fetal mortality, than girls. Late-fetal-death rates fell faster for White mothers than for those with Black or Brown skin colour, which is possibly associated with better and earlier access to quality antenatal care.^23^ In contrast, fetal mortality remained unchanged for gestations of women with Black or Brown skin colour—a finding that merits further research.

Trends over time in absolute wealth-related inequalities, expressed as differences in terms of deaths per 1000, were reduced for the three mortality indicators, particularly for infant mortality. In contrast, relative inequality—expressed as the ratios of mortality rates—remained constant. This apparent discrepancy in findings is common when rates are falling for all groups, and mortality levels among the wealthy still have scope for reduction, as was the case in 1982.^34^ In terms of absolute differences, it is quite remarkable that, as expressed by the slope index, there were 69 more deaths per 1000 at the bottom than at the top of the income scale in 1982, and this difference fell to 17 by 2015. A marked reduction in wealth-related inequalities was also observed for Brazil as a whole in the past couple of decades.^7^

The strengths of the present analyses include the population-based nature of the four cohorts and the consistency of methods used over a 33-year period. The limitations include the relatively small number of deaths, particularly fetal deaths and deaths due to specific causes. Nevertheless, the low *P*-levels for analyses where disparities were present (e.g. by income in the early cohorts) suggests that statistical power was adequate for most purposes. A second limitation includes the changes in definition over time for fetal deaths as, in 1982, only deaths to fetuses with 28 weeks’ or longer gestational age or a birthweight of 1000 g were recorded, in contrast to the later cohorts in which 20 weeks and 500 g were the cut-offs. This limitation was overcome by restricting all analyses to late-fetal deaths, complying with the 1982 definition. Fetal-mortality rates would evidently be higher had the current definition been adopted. Thirdly, information on gestational age was not comparable in the four cohorts as, in 1982 and 1993, it was based on the date on the last menstrual period and, in 2004 and 2015, it was derived primarily through obstetric ultrasound; in the present analyses, information on gestational age was only used for the definition of fetal deaths and it is unlikely that the important reduction in these rates was due to such methodological differences.

Another limitation is the information on the causes of deaths, which could only be coded in broad categories due to the lack of autopsies and to the changes in the International Classification of Diseases (ICD) codes over time; the categories used for the classification in 1982 had to be maintained for comparability.^35^Supplementary Box 1, available as Supplementary data at *IJE* online, shows the ICD9 and 10 codes. Lastly, Brazil was suffering from hyperinflation in 1993, with monthly rates close to 100% in some months, and this affected the information in family income for this cohort.

A detailed analysis of which factors contributed to the decline in mortality and reduction in absolute inequalities is beyond the scope of the present analyses. A review of child-mortality trends in Brazil as a whole suggested that progress may be explained by five sets of determinants: ‘(a) socioeconomic and demographic changes (economic growth, reduction in income disparities between the poorest and wealthiest populations, urbanization, improved education of women, and decreased fertility rates); (b) interventions outside the health sector (conditional cash transfer programs and improvements in water and sanitation); (c) vertical health programs in the 1980s (promotion of breastfeeding, oral rehydration, and immunizations); (d) creation of a tax-funded national health service in 1988 (coverage of which expanded to reach the poorest areas of the country through the Family Health Program in the mid-1990s); and (e) implementation of many national and state-wide programmes to improve child health and child nutrition.’^7^

The accompanying articles in this supplement on the Pelotas cohorts show the likely contribution of positive changes in socio-economic status, women’s education, fertility, birth intervals and access to care.^13^^,^^23^^,^^36^^,^^37^ On the other hand, there has been an increase in preterm births, which is likely to have precluded an even faster decline in mortality rates; the prevalence of low birthweight remained stable over the three decades.^14^

Substantial progress has been achieved, but current mortality levels in Pelotas remain three to four times higher than in high-income countries, whereas ethnic and social inequalities remain strong. Continued monitoring of mortality levels and inequalities is essential for overcoming this situation.

## Funding

The four cohorts received funding from the following agencies: Wellcome Trust, International Development Research Center, World Health Organization, Overseas Development Administration of the United Kingdom, European Union, Brazilian National Support Program for Centers of Excellence (PRONEX), Brazilian National Council for Scientific and Tehcnological Development (CNPq), Science and Technology Department (DECIT) of the Brazilian Ministry of Health, Research Support Foundation of the State of Rio Grande do Sul (FAPERGS), Brazilian Pastorate of the Child and Brazilian Association for Collective Health (ABRASCO).

## Pelotas Cohorts Study Group

Aluisio J D Barros,^1^ Diego G Bassani,^2^ Fernando C Wehrmeister,^1^ Helen Gonçalves,^1^ Iná S Santos,^1^ Joseph Murray,^1^ Luciana Tovo-Rodrigues,^1^ Maria Cecilia F Assunção,^1^ Mariangela F Silveira,^1^ Marlos Rodrigues Domingues^1^ and Pedro R C Hallal.^1^

^1^Federal University of Pelotas, Brazil and ^2^University of Toronto, Canada.

**Conflict of interest:** None declared.

## Supplementary Material

Supplementary Data
