# Supplementary material for: Stillbirth, newborn and infant mortality: trends and inequalities in four population-based birth cohorts in Pelotas, Brazil, 1982–2015
Source: Int J Epidemiol. 2019 Mar 18;48(Suppl 1):i54–62. doi: 10.1093/ije/dyy129 (PMC6422061; doi:10.1093/ije/dyy129)
Supplement: Supplementary Data [file dyy129_supp.zip › dyy129_Suppl_data/dyy129_Supplementary_Tables.docx]

Supplementary Table 1. Fetal mortality (gestational age ≥28 weeks) according sex, maternal skin color, and socioeconomic status in four Birth Cohorts. Pelotas, Brazil.

|  | **Fetal mortality (number of deaths)** | | | |
| --- | --- | --- | --- | --- |
|  | **1982** | **1993** | **2004** | **2015** |
| **Sex** |  |  |  |  |
| **Males** | 49 | 27 | 18 | 22 |
| **Females** | 47 | 24 | 20 | 14 |
|  |  |  |  |  |
| **Maternal skin color** |  |  |  |  |
| **White** | 76 | 35 | 22 | 16 |
| **Brown** | 21^#^ | 3 | 4 | 6 |
| **Black** |  | 17 | 15 | 14 |
|  |  |  |  |  |
| **Family income (tertiles)** |  |  |  |  |
| **Q1 (poorest)** | 70 | 17 | 14 | 19 |
| **Q2** | 18 | 20 | 21 | 12 |
| **Q3 (richest)** | 9 | 17 | 6 | 5 |
|  |  |  |  |  |
| **TOTAL** | 97 | 55 | 41 | 36 |

^#^ black and brown were combined

Supplementary Table 2. Neonatal mortality according sex, maternal skin color and socioeconomic variables in four Birth Cohorts. Pelotas, Brazil.

|  | **Neonatal mortality (number of deaths)** | | | |
| --- | --- | --- | --- | --- |
|  | **1982** | **1993** | **2004** | **2015** |
| **Sex** |  |  |  |  |
| **Males** | 68 | 46 | 33 | 21 |
| **Females** | 50 | 28 | 19 | 16 |
|  |  |  |  |  |
| **Maternal skin color** |  |  |  |  |
| **White** | 95 | 50 | 31 | 23 |
| **Brown** | 24^#^ | 6 | 4 | 5 |
| **Black** |  | 19 | 17 | 9 |
|  |  |  |  |  |
| **Family income (tertiles)** |  |  |  |  |
| **Q1 (poorest)** | 47 | 29 | 24 | 16 |
| **Q2** | 48 | 26 | 14 | 14 |
| **Q3 (richest)** | 24 | 16 | 14 | 7 |
|  |  |  |  |  |
| **TOTAL** | 119 | 75 | 52 | 37 |

^#^ black and brown were combined

Supplementary Table 3. Infant mortality according sex, maternal skin color and socioeconomic status in four Birth Cohorts. Pelotas, Brazil.

|  | **Infant mortality (number of deaths)** | | | |
| --- | --- | --- | --- | --- |
|  | **1982** | **1993** | **2004** | **2015** |
| **Sex** |  |  |  |  |
| **Males** | 118 | 65 | 47 | 29 |
| **Females** | 96 | 45 | 35 | 30 |
|  |  |  |  |  |
| **Maternal skin color** |  |  |  |  |
| **White** | 156 | 73 | 48 | 36 |
| **Brown** | 59^#^ | 8 | 7 | 8 |
| **Black** |  | 30 | 27 | 15 |
|  |  |  |  |  |
| **Family income (tertiles)** |  |  |  |  |
| **Q1 (poorest)** | 121 | 47 | 41 | 29 |
| **Q2** | 61 | 42 | 23 | 19 |
| **Q3 (richest)** | 33 | 17 | 18 | 11 |
|  |  |  |  |  |
| **TOTAL** | 215 | 111 | 82 | 59 |

^#^black and brown were combined

Supplementary Table 4. Perinatal mortality rate according to sex, maternal skin color and socioeconomic status in four Birth Cohorts. Pelotas, Brazil.

|  | **Perinatal mortality rate (1,000 total births – 95% CI)** | | | |  |
| --- | --- | --- | --- | --- | --- |
|  | **1982** | **1993** | **2004** | **2015** | **p*** |
| **Sex** | ***p= 0.725*** | ***p= 0.046*** | ***p= 0.406*** | ***p= 0.210*** |  |
| **Males** | 32.7 (26.4 - 39.0) | 25.1 (19.1 - 31.1) | 19.4 (13.7 - 25.2) | 17.8 (12.3 - 23.4) | <0.001 |
| **Females** | 31.1 (24.8 - 37.4) | 17.2 (12.3 - 22.2) | 16.1 (10.6 - 21.5) | 11.3 (6.8 - 15.8) | <0.001 |
|  |  |  |  |  |  |
| **Maternal skin color** | ***p= 0.691*** | ***p= 0.010*** | ***p< 0.001*** | ***p< 0.001*** |  |
| **White** | 31.9 (27.0 - 36.7) | 18.8 (14.7 - 23.0) | 14.1 (10.0 - 18.3) | 10.0 (6.5 - 13.5) | <0.001 |
| **Brown** | 34.2 (23.4 - 45.1) ^#^ | 38.0 (13.6 - 62.4) | 16.7 (2.2 - 31.3) | 17.6 (6.8 - 28.5) | 0.027 |
| **Black** |  | 31.9 (20.8 - 42.9) | 34.8 (22.6 - 47.1) | 33.7 (19.8 - 47.5) | 0.968 |
|  |  |  |  |  |  |
| **Family income (tertiles)** | ***p< 0.001**** | ***p= 0.039**** | ***p= 0.030**** | ***p= 0.002**** |  |
| **T1 (poorest)** | 51.2 (41.6 - 60.7) | 26.9 (18.9 - 35.0) | 22.1 (14.6 - 29.7) | 20.2 (13.1 - 27.2) | <0.001 |
| **T2** | 29.5 (22.1 - 36.9) | 22.6 (15.7 - 29.4) | 22.1 (14.5 - 29.8) | 17.0 (10.1 - 23.9) | 0.021 |
| **T3 (richest)** | 15.6 (10.2 - 21.1) | 16.5 (10.7 - 22.4) | 11.2 (5.8 - 16.7) | 6.3 (2.2 - 10.5) | 0.010 |

p-value: χ^2^ test for heterogeneity

*p-value: χ^2^ for trend

^#^black and brown were combined

Interpretation: Supplementary Table 4 shows perinatal mortality rates according to sex, maternal skin color and socioeconomic status in four cohorts. Perinatal mortality was associated with male sex, black or brown maternal skin color, and low income.

Supplementary Table 4a. Perinatal mortality according to sex, maternal skin color and socioeconomic status in four Birth Cohorts. Pelotas, Brazil.

|  | **Perinatal mortality (number of deaths)** | | | |
| --- | --- | --- | --- | --- |
|  | **1982** | **1993** | **2004** | **2015** |
| **Sex** |  |  |  |  |
| **Males** | 101 | 66 | 43 | 39 |
| **Females** | 91 | 46 | 33 | 24 |
|  |  |  |  |  |
| **Maternal skin color** |  |  |  |  |
| **White** | 157 | 77 | 44 | 31 |
| **Brown** | 37^#^ | 9 | 5 | 10 |
| **Black** |  | 31 | 30 | 22 |
|  |  |  |  |  |
| **Family income (tertiles)** |  |  |  |  |
| **Q1 (poorest)** | 104 | 42 | 32 | 31 |
| **Q2** | 59 | 41 | 31 | 23 |
| **Q3 (richest)** | 31 | 30 | 16 | 9 |
|  |  |  |  |  |
| **TOTAL** | 194 | 117 | 79 | 63 |

^#^ black and brown were combined

Supplementary Table 5. Post-neonatal mortality rate according to sex, maternal skin color and socioeconomic status in four Birth Cohorts. Pelotas, Brazil.

|  | **Post-neonatal mortality rate (1,000 total births – 95% CI)** | | | |  |
| --- | --- | --- | --- | --- | --- |
|  | **1982** | **1993** | **2004** | **2015** | **p*** |
| **Sex** | ***p= 0.887*** | ***p= 0.702*** | ***p= 0.565*** | ***p= 0.180*** |  |
| **Males** | 16.5 (11.9 - 20.9) | 7.3 (4.0 - 10.6) | 6.4 (3.0 - 9.7) | 3.7 (1.1 - 6.3) | <0.001 |
| **Females** | 16.0 (11.4 - 20.5) | 6.4 (3.4 - 9.5) | 7.9 (4.0 - 11.7) | 6.6 (3.2 - 10.1) | 0.001 |
|  |  |  |  |  |  |
| **Maternal skin color** | ***p< 0.001*** | ***p= 0.136*** | ***p= 0.123*** | ***p= 0.253*** |  |
| **White** | 12.6 (9.4 - 15.7) | 5.7 (3.4 - 8.0) | 5.5 (2.9 - 81.1) | 4.2 (1.9 - 6.5) | <0.001 |
| **Brown** | 33.0 (22.3 - 43.7)^#^ | 8.5 (0.0 - 20.4) | 10.2 (0.0 - 21.6) | 5.3 (0.0 - 11.4) | <0.001 |
| **Black** |  | 11.5 (4.7 - 18.3) | 11.8 (4.5 - 19.1) | 9.4 (1.9 - 16.9) | <0.001 |
|  |  |  |  |  |  |
| **Family income (tertiles)** | ***p< 0.001**** | ***p< 0.001**** | ***p= 0.004**** | ***p= 0.029**** |  |
| **T1 (poorest)** | 37.7 (29.3 - 46.1) | 11.7 (6.3 - 17.0) | 11.9 (6.3 - 17.5) | 8.6 (3.9 -13.2) | <0.001 |
| **T2** | 6.6 (3.0 - 10.1) | 8.9 (4.6 - 13.2) | 6.5 (2.3 - 10.8) | 3.7 (0.5 - 7.0) | 0.286 |
| **T3 (richest)** | 4.6 (1.6 - 7.5) | 0.6 (0.0 - 1.6) | 2.8 (0.0 - 5.6) | 2.8 (0.0 - 5.6) | 0.498 |

p-value: χ^2^ test for heterogeneity

*p-value: χ^2^ for trend

^#^black and brown were combined

Interpretation: Post-neonatal mortality rates were similar for boys and girls in all four cohorts. Except for 1982 when infants born to white mothers had lower rates, there was no statistical evidence of ethnic disparities in the other cohorts. In all cohorts, post-neonatal mortality was inversely related to family income. P levels have to be interpreted with due caution due to the small number of deaths in some categories.

Supplementary Table 5a. Post-neonatal mortality according to sex, maternal skin color and socioeconomic status in four Birth Cohorts. Pelotas, Brazil.

|  | **Post-neonatal mortality (number of deaths)** | | | |
| --- | --- | --- | --- | --- |
|  | **1982** | **1993** | **2004** | **2015** |
| **Sex** |  |  |  |  |
| **Males** | 50 | 19 | 14 | 8 |
| **Females** | 46 | 17 | 16 | 14 |
|  |  |  |  |  |
| **Maternal skin color** |  |  |  |  |
| **White** | 61 | 23 | 17 | 13 |
| **Brown** | 35^#^ | 2 | 3 | 3 |
| **Black** |  | 11 | 10 | 6 |
|  |  |  |  |  |
| **Family income (tertiles)** |  |  |  |  |
| **Q1 (poorest)** | 74 | 18 | 17 | 13 |
| **Q2** | 13 | 16 | 9 | 5 |
| **Q3 (richest)** | 9 | 1 | 4 | 4 |
|  |  |  |  |  |
| **TOTAL** | 96 | 36 | 30 | 22 |

^#^ black and brown were combined
