# Supplementary material for: Stillbirth, newborn and infant mortality: trends and inequalities in four population-based birth cohorts in Pelotas, Brazil, 1982–2015
Source: Int J Epidemiol. 2019 Mar 18;48(Suppl 1):i54–62. doi: 10.1093/ije/dyy129 (PMC6422061; doi:10.1093/ije/dyy129)
Supplement: Supplementary Data [file dyy129_supp.zip › dyy129_Suppl_data/dyy129_Supplementary_Box_S1.docx]

Supplementary Box 1. International Classification of Mortality (ICD9 and ICD10) used in the paper.

|  | ICD9 | ICD10 |
| --- | --- | --- |
| Perinatal | 760-779.9 | P00-P96 |
| Congenital Malformations | 740-759.8 | Q00-Q99 |
| Diarrhea | 001-009.3 | A00-A09 |
| Respiratory Infections | 460-519.9 | J00-J86 |
| Other infections | 090 | B90-B99 |
| Other causes or ill-defined | 780-799.9 | R00-R99 |
