# Supplementary material for: Stillbirth, newborn and infant mortality: trends and inequalities in four population-based birth cohorts in Pelotas, Brazil, 1982–2015
Source: Int J Epidemiol. 2019 Mar 18;48(Suppl 1):i54–62. doi: 10.1093/ije/dyy129 (PMC6422061; doi:10.1093/ije/dyy129)
Supplement: Supplementary Data [file dyy129_supp.zip › dyy129_Suppl_data/dyy129_Supplementary_Table_S6.docx]

Supplementary Table 6. Prevalence ratios (PR) for neonatal and infant mortality in the 1993, 2004 and 2015 cohorts, using the 1982 cohort mortality rate as the reference. Crude and gestational-age adjusted results.

|  | Neonatal mortality | | Infant mortality | |
| --- | --- | --- | --- | --- |
| Cohort | PR (95% CI)  Crude | PR (95% CI)  Adjusted* | PR (95% CI)  Crude | PR (95% CI)  Adjusted* |
| 1982 | 1.0 (reference) | 1.0 (reference) | 1.0 (reference) | 1.0 (reference) |
| 1993 | 0.71 (0.53; 0.94) | 0.49 (0.34; 0.71) | 0.58 (0.46; 0.73) | 0.44 (0.33; 0.59) |
| 2004 | 0.61 (0.44; 0.84) | 0.51 (0.36; 0.72) | 0.53 (0.41; 0.68) | 0.46 (0.36; 0.61) |
| 2015 | 0.43 (0.30; 0.62) | 0.33 (0.22; 0.48) | 0.38 (0.29; 0.50) | 0.30 (0.23; 0.41) |

(*) Adjusted for gestational age (<37; 37-38;3 9-41; ≥42 weeks).
